# Building resilience from the ground up

**DOI:** 10.1111/disa.12345

**Published:** 2019-04-04

**Authors:** Emily Wilkinson, Caroline King‐Okumu

**Affiliations:** ^1^ Senior Research Fellow, Risk and Resilience Programme, Overseas Development Institute United Kingdom; ^2^ International Development Opportunities Manager, Centre for Ecology and Hydrology United Kingdom

## Introduction

Climate extremes and longer‐term climate change impacts threaten the achievement of development goals (Damania et al., 2017; IBRD, 2018). In 2030, up to 319 million extremely poor people will be living in the 45 countries most exposed to floods, droughts and heat extremes (Shepherd et al., 2013). According to the recent Intergovernmental Panel on Climate Change report on 1.5°C global warming, a 2°C increase in temperature will double^1^ the number of people exposed to drought through water stress (IPCC, 2018). To eradicate extreme poverty by 2030, development cooperation and domestic action in the developing world is increasingly concerned with building resilience to these climate hazards (SDG Goal 1.5).

Amid lively critical debates, the resilience‐building agenda has sparked a proliferation of projects in recent years. Resilience programmes are being implemented in some of the most climate‐vulnerable, institutionally fragile and unstable settings around the world. Often, they focus on improving people's access to climate and weather information, resources or markets, helping them plan ahead and navigate environmental change and uncertainty in the future. Importantly, resilience‐building is anticipated to give greater agency to vulnerable people and produce more co‐benefits or ‘dividends’ than conventional international development approaches (Rodin, 2014; Tanner et al., 2015a, 2015b; Bond et al., 2017; Cabot Venton, 2018; Cabot Venton et al., 2012).

Building resilience from the ground up is critical because of the context‐specific nature of climate change and disaster impacts and the need to ensure the engagement of vulnerable groups. The five‐year, £100m UK Department for International Development (DFID) programme on Building Resilience and Adaptation to Climate Extremes and Disasters (BRACED) is one example of this intervention logic. Alongside the implementation of 15 projects in 13 countries, research and monitoring and evaluation, knowledge‐sharing activities that have taken place under BRACED present a unique opportunity to learn about how poor people and communities deal with climate shocks and other hazards in different contexts, their vulnerabilities and the kinds of interventions that can help strengthen their resilience. Similarly, the Action on Climate Today (ACT) programme funded by DFID for five years to provide technical and financial support to governments across five South Asian countries had a lesson‐learning function to share experiences and knowledge across the programme and with the outside world.

This special issue of *Disasters* reflects on resilience‐building supported via BRACED and ACT in some of the world's most climate‐vulnerable countries and contexts. These programmes have focused on scaling up action to build resilience, ^2^ principally through the expansion and replication of good practices by influencing government policies, plans and investments. The papers provide insights that are each grounded in different contexts and understandings of local realities and the factors that support and undermine people's resilience. The BRACED articles emphasise the importance of this ground‐level engagement. They also highlight a range of different opportunities for intervening in the broader social structures and decision‐making processes that shape these local realities. The focus of the ACT article is explicitly and exclusively concerned with national and local government policy‐making and how this can be influenced.

Each of the seven papers selected for this special issue was written by teams of researchers and practitioners engaged in the BRACED and ACT programmes, based in the Global South and North in a range of country contexts from the Sahel to Southeast Asia. Each brings a different perspective on the significance and operationalisation of efforts to build resilience to climate extremes and disasters. The contributing authors describe resilience‐building at different scales, for different types of projects and interventions: from gender‐differentiated perspectives within households in Ethiopia, Burkina Faso and Chad (Le Masson et al., 2019; McOmber et al., 2019), to devolved community planning and financing in Mali and Senegal (Beauchamp et al., 2019); from sector‐wide agricultural extension support in Sudan (Young and Ismail, 2019), to early warning systems in Ethiopia and Nicaragua (Ewbank et al., 2019), national social protection programmes in Ethiopia (Ulrichs et al., 2019) and advocacy for mainstreaming into government policy in South Asia (Tanner et al., 2019).

It is important to understand that the projects of the BRACED and ACT programmes, which inspired the articles in this special issue, did not and could not invest in long‐term studies of the kind that could test the hypotheses of the resilience‐building programmes – that, through these sets of interventions, people's resilience could be enhanced and hence the negative impact of climate extremes and longer‐term climate change be reduced. This was because of the brief implementation time frames of both programmes. This is a common challenge with project‐based interventions, where the assessment of potential impacts needs to be done simultaneously with the investments intended to achieve these impacts. As such, these articles are not based on routine evidence of results produced through monitoring, learning and evaluation activities, but rather are selected expert reflections on the projects. They are intended for scientific peer review and journal publication, not project evaluation.

The articles present rich and detailed descriptions and reflections from their respective fields and contexts, which we do not exhaustively or definitively summarise in this overview. Rather, this overview paper aims to gently pique the curiosity of readers, and to reflect briefly on the critical questions that the articles help address, which have hereto remain largely unresolved in resilience debates. We select and draw out insights from this special issue that feed into these debates, and highlight their significance for the wider community of humanitarian and development practitioners.

Following a brief overview of how the term ‘resilience’ is being used in each of the articles, we then examine how each has approached the challenge of understanding and measuring bottom‐up interventions. We consider the multiple benefits or resilience dividends that make some of these projects unique, and reflect on what the authors consider are the prospects for effecting deeper structural, or transformative changes.

## Understanding resilience on the ground

Resilience is a curiously amorphous concept taken from the discipline of ecology to denote a self‐sustaining dynamic capability (IPCC, 2014, n.p.):

*… the capacity of social, economic and environmental systems to cope with a hazardous event or trend or disturbance, responding or reorganising in ways that maintain their essential function, identity and structure, while also maintaining the capacity for adaptation, learning and transformation*.

DFID (2014a, p. 4), on the other hand, focuses on the attributes of individuals, defining resilience as:

*… a composite attribute possessed by each individual, that represents their ability to anticipate, avoid, plan for, cope with, recover from and adapt to (climate related) shocks and stresses. Improved resilience means that an individual is better able to maintain or improve their well‐being despite being exposed to shocks and stresses*.

Diverse references are used within the BRACED and ACT programmes in defining resilience, including Béné et al. (2012), DFID (2014a), and Bahadur et al. (2015), but all articles in this special issue converge around the general view that improving human capacities and decision‐making is the key to building resilience to climate extremes and disasters.

At the individual level, for example, Le Masson et al. (2019, p. S184) understand resilience to be:

*… the ability of women and men to exercise their rights and improve their well‐being despite traumatic events, stresses and uncertainty*.

Le Masson et al. (2019) and McOmber et al. (2019) allude to development failures of the past that have disadvantaged particular individuals and social groups, heightening their vulnerability to disasters and climate change impacts. McOmber et al. (2019) observe that focusing on the stability of a system as a whole can obscure the structural and historical drivers of vulnerability and the power imbalances that arise internally at different scales. Le Masson et al. (2019, p. S179) observe that imbalanced decision‐making processes undermine adjustments that could have been made in anticipation of or response to climate and related shocks and stresses, and argue that:

*Development programmes that aim to build resilience need at a minimum to avoid perpetuating gender inequalities, and can support several processes of social change to tackle violence and build resilience at individual, household and community levels*.

They point out that violence against women and girls undermines the capacities of survivors to cope with crises by exercising their rights and improving their well‐being. As a result, a transformative approach is needed to address underlying structural inequalities, discriminatory practices and gender norms, including those that result in gender‐based violence.

Ulrichs et al. (2019) observe that applying ‘resilience thinking’ to social protection requires consideration of issues that do not normally receive much attention in this field of development practice. It pushes those designing social protection programmes to look beyond helping people so they can absorb shocks, towards a more anticipatory approach. Social protection programmes are undergoing changes to help beneficiaries anticipate immediate shocks and absorb some of their impacts, and are beginning to explore opportunities to help people to adapt. If resilience‐building entails strengthening the ‘3As’ of adaptive, absorptive and anticipatory capacity, as described by Bahadur et al. (2015), then, according to Ulrichs et al. (2019), shock‐responsive social protection programmes are less suitable for strengthening adaptive capacity. However, they note that, nonetheless, social protection programmes could work well in communities able to drive their own adaptation agendas.

Young and Ismail (2019) see resilience‐building as an alternative approach, contrasting with international humanitarian discourse and media narratives focused on Darfur during the past 15 years that have polarised divisions between pastoralists and farming communities. In contrast, they consider resilience‐building approaches inspired by ecologists to be better able to engage with the complexity of the long‐term symbiotic relationship that exists between communities and their production systems. Young and Ismail (2019, p. S258) argue that:

*Darfuri producers, whether in farming or pastoralism, are specialised to take advantage of extreme environmental variability. The roots of resilience of Darfur livelihood systems rest in the continuity and integration of these livelihood specialisations, as part of a regional livelihood system*.

Similarly, Beauchamp et al. (2019) engage with the context‐dependent nature of resilience. They highlight the important differences between donor understandings of resilience and those of vulnerable people in the Sahel, and emphasise and explore subjective dimensions of resilience with local community members.

Ewbank et al. (2019) highlight the mediating role of social support structures, helping build resilience by preventing hazards from becoming disasters and therefore strengthening the resilience of farming communities. Their article illustrates how disseminating information about the weather and climate through these systems helps people in farming communities access and use it to take better decisions. This illustrates a practical case where well‐designed and communicated access to previously unavailable information enables people to become more resilient.

Tanner et al. (2019) emphasise that building resilience is different from business‐as‐usual development, but that adaptation and development processes are inextricably linked. As such, climate change is increasingly being integrated or ‘mainstreamed’ into development planning and sectoral decision‐making, in order that adaptation measures become ‘part of a broader suite of measures within existing development processes and decision cycles’ (OECD, 2009).

## Understanding the value of bottom up interventions

The authors of these special issue articles note that understanding and demonstrating changes in resilience is challenging. As donors and practitioners continue to search for generic and globally applicable measures of resilience—to help prioritise actions, increase accountability, guide programming and scale up investments—so too do academic critiques continue to grow. Scholars see resilience as complex and contested, and there is little consensus on evaluating resilience interventions (Levine, 2014; Schipper and Langston, 2015; Sharifi, 2016; Otsuki et al., 2017; Quandt, 2018; Hallegatte and Engle, 2018). With such a broad range of issues and agendas that need addressing to help people deal with risks and uncertainties in their environment, it is unlikely that any metric, however broad and complex, will do. Rather, the articles in this special issue demonstrate how multiple factors shape people's ability to understand the risks they face, actively manage them and thrive in some extremely hostile and insecure environments.

Commentators are often reluctant to reduce the multifaceted concept of resilience to any fixed objective measure. Following Bahadur et al. (2015), most of the contributions in this special issue focus on context‐specific measurements—the characteristics of the households and communities that best capture their ability to deal with shocks and can be used as proxies for resilience (see Frankenberger and Nelson, 2013). For example, Ulrichs et al. (2019) consider social protection to have contributed to resilience by helping people meet food needs and not deplete assets. Young and Ismail (2019) also focus on familiar measures of resilience in Darfur, in terms of continued agricultural productivity. These are not *new* measurements—not in development programming—but they are used in new ways.

Beauchamp et al. (2019) set out to test the hypothesised relationships between resilience, food security and other variables that capture different dimensions and perspectives of the concept of resilience. Using a survey of household perceptions, they find that many of the indicators suggested for measuring progress against DFID's Key Performance Indicator KPI4 correlate with the characteristics of resilience identified by beneficiaries of the BRACED Decentralising Climate Finance project (that is, self‐assessed resilience). Their research suggests broad alignment between attributes of self‐assessed resilience and a self‐reported food security proxy. Yet differing patterns in the results comparing self‐assessed resilience and food security in different locations suggest there is more complexity than one variable can capture.

Several of the contributions draw on an approach to assessing resilience outlined in DFID's KP14 guidance (DFID, 2014a), focussing on individual well‐being, exploring multiple aspects of resilience and what different levels of resilience and well‐being look like. For example, Beauchamp et al. (2019) focus on the resilience features linked to livelihoods and agro‐ecological zones, as identified by individuals, and explore investment options to improve resilience and well‐being (see specific examples and further description in Keita and Koulibaly, 2016).

The use of the BRACED Participatory Approach (BRAPA) is also described and discussed by both Ewbank et al. (2019) and McOmber et al. (2019) as a means to measure changes in resilience. These authors anticipate that BRAPAs could even provide baselines for future monitoring. To our knowledge, rapid assessment techniques such as BRAPA are not often used in this way. McOmber et al. (2019) also noted the importance of qualitative tools such as focus group discussions and open‐ended interviews to assess resilience interventions; however, this approach is very time intensive. Also, results and much of the analysis could not be properly input into the projects’ structure owing to tight deadlines. They observed that the approach also had other limitations:

*While some of the questions were gender‐disaggregated and helped to reveal a quantitative assessment of gendered access to information, the tool did not allow an understanding of the underlying mechanisms that prevented women having equal access to and utilisation of climate information and subsequent decision‐making dynamics* (McOmber et al., 2019, pp. S201–S202).

McOmber et al. (2019) stress in particular the difficulties in operationalising, framing, measuring and evaluating the impact of activities aimed at reducing the power inequalities that undermine women's resilience. Their article highlights the need to create finer‐tuned mechanisms and tools for M&E and learning processes that can more fully capture these nuanced and subtle experiences of change.

One option beyond the resilience measurement impasse suggested by some of the authors is to move away from specific metrics towards more generic and globally comparable resilience dividends (see, for example, Rodin, 2014; Bond et al., 2017). The costs of building resilience—be they public or private expenditures—need to be considered in relation to the potential multiple benefits of these multi‐faceted projects, which not only help individuals and communities survive hazards but also generate other co‐benefits for society, the local economy and the environment, enhancing productivity and well‐being—all of which add to the overall value of resilience initiatives (Tanner et al., 2015a, 2015b; King‐Okumu et al., 2017, 2018; Coulibaly et al., 2018). Ewbank et al. (2019), for example, explore the value of resilience‐building interventions that reduce input costs, increase productivity and reduce damage from disasters.

Ewbank et al. (2019) note the methodological challenges of measuring losses that are *not* incurred as a result of successful resilience‐building. They do not look into the potential for using simulation tools to model and compare different scenarios, and the BRACED programme has not promoted the use of such tools—they are not mentioned in DFID KP14 guidance (DFID, 2014a). However, during the programme some exploration was conducted of the potential to use hydro‐meteorological decision support tools in future to generate quantitative assessments (as described in King‐Okumu et al., 2017). It is important to emphasise that such assessments would still need to be accompanied by continuation of the in‐depth qualitative evaluation work that the BRACED programme has supported. Interestingly, many of the context‐specific indicators explored in the articles so far would lend themselves relatively easily to an economic valuation.

The studies described in this special issue were conducted in parallel with the implementation of programmes funded through BRACED. With hindsight, there will be increased opportunities to observe the hazards and impacts that did and did not occur, and to identify signs and perceptions of changes and longer‐term shifts. In some instances, the use of national statistics and databases could support such analyses, as recently attempted in the Horn of Africa by Cabot Venton (2018). Although the studies described in this special issue were not able to include ‘control’ cases, as time has advanced, retrospective description of carefully selected ‘untreated’ areas and cases could also now be explored. This may help support assessments of effects achieved on resilience.

## Effecting deeper structural changes

A critical question for these DFID and ACT interventions is whether they are able to bring about deeper structural or transformative changes during a short implementation period. For DFID, initiatives are transformative if they are catalytic, achieve impact at scale and produce sustainable outcomes (DFID, 2014b). These three are intrinsically linked. Catalytic effects imply the ability to leverage wider change, including the replication and financing of similar approaches by others. Linked to this, initiatives are sustainable if beneficial effects can continue beyond donor funding by producing shifts in policy, regulations and behaviour. Achieving impact at scale, a particular focus of the BRACED programme, occurs when interventions are used at a greater scale or in integrated combinations with much larger effects than before (Kates et al., 2012). For Bahadur et al. (2015, p. 41): ‘The scale of impacts may be measured in terms of the outcomes achieved in relation to the magnitude of resource inputs'.

Many of the articles focus on influencing public officials to bring about transformational changes. Tanner et al. (2019), for example, focus on scaling up via the political processes of influencing government policy planning and sectoral decision‐making on climate adaptation. Young and Ismail (2019) document and describe resilience in Darfur, and how local actors are linking to and engaging with government and wider civil society networks, resulting in a shared understanding of the experiences of local people and their ability to manage variability.

To tackle violence against women and girls, Le Masson et al. (2019) also recommend working with government representatives to share information on the rights of citizens and spread messages to change attitudes towards inequalities. Pragmatically, they explain that this is effective because information is conveyed by a man, in the local language and based on the law and on religious texts. This is a less direct pathway to effecting change and empowering women and girls than one that would immediately encourage women to raise their voices directly—since it works with the existing status quo. The article recommends creating awareness and working gently through other programmes that people are interested in until they are ready to address the problem more directly.

Ulrichs et al. (2019) describe how social protection has been adapted to contribute to (national) drought relief and preparedness. Payments can be triggered and scaled up pre‐emptively by linking social protection to early warning systems, helping vulnerable households *before* they are forced to sacrifice their assets. Ulrichs et al. (2019) observe that the emphasis from within social protection is largely on programmes that specifically aim to address climate risks by either (1) scaling up in response to a short‐term shock episode or (2) incorporating more complex elements beyond the transfer, such as asset‐building through public works or savings and loans to enable households to build assets and transform livelihoods (described as ‘social protection plus', or productive safety net programmes). The authors also propose a third way that social protection could build anticipatory capacity, through contingency financing, pre‐registration of households at risk of exposure to shocks, etc., and tackling the vulnerabilities that reinforce the negative impacts of shocks.

Like Le Masson et al. (2019), Ulrichs et al. (2019) also caution against pushing too hard and trying to pursue structural changes too quickly within a short project time frame. A premature focus on expanding the functions and technical scope of programmes, and the addition of auxiliary features, can be ineffective and even counter‐productive if the commitment to long‐term financial and technical support is not guaranteed (either by the government or by development partners). Both Ulrichs et al. (2019) and Ewbank et al. (2019) focus on positive outcomes that have gradually emerged from the Productive Safety Nets Programme in Ethiopia since 2005, showing that resilience‐building is a long‐term process. Ewbank et al. (2019) compare early achievements in Kombolcha, Ethiopia, to those obtained in Nicaragua following six years of similar resilience‐building. In light of this, they recommend *persistent* support for early warning, early action and post‐drought recovery in order to scale up successfully.

Persistent support and upscaling advocated by Ewbank et al. (2019, p. S282) could be undertaken using climate finance at either global, national or community levels, but should always focus on delivering resilience on the ground, with vulnerable communities taking the lead:

*Drought resilience for the most vulnerable needs long‐term and consistent support to be built and maintained by all stakeholders, including climate service providers, local government and civil society. As climate change intensifies future droughts, the importance of this community‐based anticipatory approach will grow*.

Both Ewbank et al. (2019) and Beauchamp et al. (2019) describe the potential of scaling up by using resources from global climate funds, rather than individual national governments. Both speak to the need and opportunity to pilot and put in place effective M&E systems for these funds. This could be a strategic route to upscaling because, in theory, such evaluations should direct the next round of interventions. In light of this expectation, Beauchamp et al. (2019) set out to mirror the practical realities shaping the implementation of a large multi‐year M&E system, and speak to an increasing debate at the international level about aggregating programme results and national progress towards targets such as the adaptation goal (Craft and Fisher, 2018).

Further insights on the opportunities for learning as an essential and transformative part of resilience‐building are offered by McOmber et al. (2019). They explore how gender‐transformative approaches can be effectively mainstreamed into development programming learning and feedback loops. They agree with Le Masson et al. (2019) that gender‐transformative approaches are a long‐term and ambitious endeavour, and one that exceeds the scope and timeline of the BRACED programme. Still, they emphasise the achievements that have been made through the creation of spaces and methods under the BRACED programme for deconstructing and challenging systems of oppression. They consider that these ‘facilitate the negotiation and construction of new social norms of agency necessary for responding to the emerging environmental challenges of our time'.

## Overview of insights from the ground up

The articles provide examples of what resilience means in different social and environmental contexts. The contributing authors describe resilience‐building from the perspectives of different projects and at different scales, yet a number of consensus points emerge. First, building resilience from the ground up can and should look very different. Second, measurable results from resilience‐building can and have been identified within a relatively short time frame using either context‐specific or generic measures, or, ideally, a combination. Third, given the need for evidence and a better understanding of what these resilience approaches can offer, further investigation of resilience dividends is needed after these programmes have finished. Ideally, this research would be repeated after 10 years.

This collection of articles demonstrates that programmes such as BRACED and ACT can make some inroads in a short time into tackling some structural causes of vulnerability, as well as more proximate issues of exposure and livelihood security. The next step for scaling up successes should not be to wait for another similar programme and follow‐on funding; rather, project partners should seek to enable national policy‐makers, regional agencies, civil society groups and financiers to take ownership of successes achieved on the ground, and seek ways to replicate them.

For the international disaster risk reduction community, the articles reaffirm the case for investing before disasters happen. They also demonstrate the importance of accompanying resilience‐building interventions with research, learning and knowledge‐sharing. Researchers can work with project teams to help capture the local dynamics and factors that undermine people's resilience so they can tailor interventions to address these. They can also provide analysis of the broader social structures and decision‐making processes that are shaping these local realities. These insights are critical for international development agencies, including DFID, as they continue to develop resilience‐building programmes. They are also relevant to the multitude of national and local level actors who work within and alongside such programmes, who can seek to maximise the multiple resilience dividends from these interventions during their implementation and following their completion.

## Endnotes

1 In comparison with a 1.5°C warming scenario.

2 See definition in Carter et al. (2018).
